# Construction of a Novel Immune-Related lncRNA Pair Signature with Prognostic Significance for Kidney Clear Cell Renal Cell Carcinoma

**DOI:** 10.1155/2021/8800358

**Published:** 2021-09-01

**Authors:** Jian-Xuan Sun, Qi-Dong Xia, Chen-Qian Liu, Jin-Zhou Xu, Yang Xun, Jun-Lin Lu, Jia Hu, Shao-Gang Wang

**Affiliations:** Department and Institute of Urology, Tongji Hospital, Tongji Medical College, Huazhong University of Science and Technology, No. 1095 Jiefang Avenue, 430030 Wuhan, China

## Abstract

**Background:**

Renal cell carcinoma (RCC) is one of the most common aggressive malignant tumors in the urinary system, among which the clear cell renal cell carcinoma (ccRCC) is the most common subtype. The immune-related long noncoding ribonucleic acids (irlncRNAs) which are abundant in immune cells and immune microenvironment (IME) have potential significance in evaluating the prognosis and effects of immunotherapy. The signature based on irlncRNA pairs and independent of the exact expression level seems to have a latent predictive significance for the prognosis of patients with malignant tumors but has not been applied in ccRCC yet.

**Method:**

In this article, we retrieved The Cancer Genome Atlas (TCGA) database for the transcriptome profiling data of the ccRCC and performed coexpression analysis between known immune-related genes (ir-genes) and lncRNAs to find differently expressed irlncRNA (DEirlncRNA). Then, we adopted a single-factor test and a modified LASSO regression analysis to screen out ideal DEirlncRNAs and constructed a Cox proportional hazard model. We have sifted 28 DEirlncRNA pairs, 12 of which were included in this model. Next, we compared the area under the curve (AUC), found the cutoff point by using the Akaike information criterion (AIC) value, and distinguished the patients with ccRCC into a high-risk group and a low-risk group using this value. Finally, we tested this model by investigating the relationship between risk score and survival, clinical pathological characteristics, cells in tumor immune microenvironment, chemotherapy, and targeted checkpoint biomarkers.

**Results:**

A novel immune-related lncRNA pair signature consisting of 12 DEirlncRNA pairs was successfully constructed and tightly associated with overall survival, clinical pathological characteristics, cells in tumor immune microenvironment, and reactiveness to immunotherapy and chemotherapy in patients with ccRCC. Besides, the efficacy of this signature was verified in some commonly used clinicopathological subgroups and could serve as an independent prognostic factor in patients with ccRCC.

**Conclusions:**

This signature was proven to have a potential predictive significance for the prognosis of patients with ccRCC and the efficacy of immunotherapy.

## 1. Introduction

Kidney cancer is one of the 10 most common cancers around the world, accounting for about 2% of all global cancer cases, and the number of cases is rising year by year [1]. In the United States, the expected new cases and deaths caused by malignant tumors happening in the kidney and renal pelvis are 73,750 and 14,830, respectively, in 2020 [2]. In Europe, the estimated incidence and mortality for kidney cancer are 99,200 and 39,100 in 2018 [3]. As for Chinese patients, the data is estimated to be 66,800 and 23,400, respectively, in 2015 [4]. Renal cell carcinoma (RCC), which originates from the renal epithelium, accounts for >90% of the malignant tumors happening in the kidney and has numerous histological subtypes, among which clear cell renal cell carcinoma (ccRCC) is the most common and accounts for about three quarters of all cases [5]. Localized ccRCC can have a relatively good prognosis through surgery, but the prognosis for metastatic ccRCC is poor and conventional chemotherapy usually has no effect. However, the development of targeted therapies and immunotherapy are benefiting more and more patients over the last decades.

In United States and European Union, targeted agents directed at vascular endothelial growth factor (VEGF) and its receptors (VEGFRs) such as sorafenib, axitinib, sunitinib, lenvatinib, pazopanib, and cabozantinib [6–10] are regarded as the first-line and second-line medicine for metastatic RCC. Other targeted agents, such as everolimus and temsirolimus, inhibitors of mTOR signaling, are proven effective for patients with poor prognosis. As for immunotherapy, T cell immune checkpoint inhibitors are also very popular nowadays. Antibodies which inhibit programmed cell death protein 1 ligand 1 (PDL1), programmed cell death protein 1 (PD1), and T cell checkpoint cytotoxic T-lymphocyte-associated protein 4 (CTLA4) are thought to relieve T cells from inhibition in the tumor microenvironment (TME) and reactivate their function in tumor killing. Nivolumab is proven to have longer overall survival and fewer adverse reactions compared with everolimus among patients with RCC who have failed previous treatment in the CheckMate 025 clinical trial [11]. In the future, the combination of targeted therapy and immunotherapy may tremendously improve the patients' prognosis in metastatic RCC [1].

Long noncoding RNAs (lncRNAs) are a series of RNAs transcribed from the human genome, which are incapable of coding peptide sequences and are larger than 200 nt in length. Nowadays, more and more functions of lncRNAs have been discovered, such as regulating gene expression; posttranscriptional modification and splicing; translation; interaction between DNA, RNA, and protein; protein modification; and cell signaling pathways [12]. Thus, they are crucial to cell growth, differentiation, and development. The occurrence and development of many diseases are tightly connected with lncRNAs, among which cancers have attracted the most attention. lncRNAs such as PVT1, LET, HOTAIR, NBAT1, GAS5, CADM-AS1, linc00963, RCCRT1, SPRY4-IT1, and HIF1A-AS seem to take part in the tumorigenesis and development of renal cancer [13]. Recent evidences indicate that lncRNAs play a crucial role in both the innate and adaptive immune systems, such as immune cell lineage development and immune cell activation, and they may affect the tumor immune cell microenvironment by regulating tumor immune cell infiltration [14]. For example, lncRNA SATB2-AS1 appears to have the ability to regulate the proportion and density of immune cells and the expression of TH1-type chemokines in the tumor microenvironment of colorectal cancer, thus inhibiting tumor metastasis and affecting the prognosis of patients [15]. Therefore, signatures connected with the immune cell microenvironment and tumor immune cell infiltration provided by lncRNAs may have a significant function in predicting the diagnosis and prognosis of tumor and will help in choosing the appropriate treatment. Zhu et al. have constructed an eight-lncRNA signature to evaluate the response to immune checkpoint inhibitors in patients with esophageal squamous cell carcinoma [16]. Moreover, Wang et al. established a prognostic signature based on four immune-related differentially expressed lncRNAs (DElncRNAs) for lung adenocarcinoma [17]. As for renal clear cell carcinoma, a novel five immune-related lncRNA signature has been constructed by Sun et al. and is proven to have a predictive significance for the prognosis [18].

However, all models previously established are based on one biomarker and its expression level. It has been reported that the combination of two biomarkers will provide a predictive model with higher accuracy [19]. Hong et al. have constructed a novel irlncRNA signature based on the combination of two irlncRNAs and not dependent on their expression levels in hepatocellular carcinoma, and this model has displayed good predictive significance and may help screen out patients that can benefit from immunotherapy [20]. Thus, in this article, we established a similar irlncRNA pair signature independent of specific expression levels in ccRCC. Then, we applied this model in patients suffering from ccRCC and assessed its predictive value and diagnostic effectiveness. Ultimately, we estimated the predictive power of the tumor immune microenvironment and analyzed the interaction between this risk model and chemotherapeutics.

## 2. Materials and Methods

### 2.1. Data Sources

The transcriptome profiles and corresponding clinical characteristics of kidney clear cell carcinoma were downloaded from the KIRC project in TCGA_GDC (https://portal.gdc.cancer.gov/). The immune-related gene list was retrieved from the IMMPORT database (https://www.immport.org/home). The gene annotation file used to annotate gene as protein-coding or lncRNA was downloaded from the ENSEMBL database (https://asia.ensembl.org/index.html).

### 2.2. Identification of Immune-Related lncRNA Pairs

We firstly extracted two transcriptional expression atlases, including the expression of immune genes and the expression of lncRNAs; then, the Pearson correlation test was used to identify immune-related lncRNA. Notably, those lncRNAs with absolute correlation coefficient ≥ 0.8 and adjusted *p* value < 0.001 were considered as immune-related lncRNAs for further analysis. 0.8 ≤ correlation coefficient < 1 is considered to be a very strong correlation in statistics. Adjusted *p* value < 0.001 can screen out lncRNAs with more significant correlations. Subsequently, we conducted differential expression analysis to identify those differentially expressed immune-related lncRNAs with logFC ≥ 1 and FDR < 0.05. ∣LogFC | ≥1 refers to genes which are differentially expressed twice or more between normal tissues and tumor tissues. The *p* value adjusted by FDR < 0.05 can further screen out lncRNAs that are differentially expressed between tumor and normal tissues. It is worth mentioning that logFC ≥ 1 and FDR < 0.05 are commonly used criteria for identifying differentially expressed genes in differential expression analysis [21]. Following this, we performed cyclical single-paired analysis on these lncRNAs to define immune-related lncRNA pairs. Moreover, only the pair ratio was stable between 20% of the patients and 80% of the patients, the lncRNA pairs were considered suitable for further analysis, and we got a matrix with 0 or 1 that for a lncRNA pair A | B, 0 means in this sample A expression was lower than B, 1 means in this sample A expression was higher than B. If the value of the lncRNA pair is 0 or 1 in all people, that is, the expression levels of two lncRNAs in all people are the same, then there is no need to pair and construct a predictive model. Therefore, here we will consider the lncRNA pairs that can obtain stable values in 20%-80% of the patients as suitable lncRNA pairs for further analysis [20].

### 2.3. Development of lncRNA Pair-Based Prognostic Signature

Having identified all immune-related lncRNA pairs, we performed uni-Cox regression for each lncRNA pair to filter those lncRNA pairs with prognostic value *p* < 0.05. Then, we conducted LASSO regression to avoid overfitting and acquire appropriate variables. Subsequently, the multivariate Cox regression was used to construct a survival-predicting signature, and each sample acquired a risk score according to the formula developed by the multivariate Cox regression as follows: riskScore = ∑_*i*=1_^*N*^(value(*i*)∙coef(*i*)), where, *N* means the total number of lncRNA pairs included in this signature, value(*i*) means the matrix value of this lncRNA that is either 0 or 1, and coef(*i*) means the coefficient of this lncRNA pair.

### 2.4. Validation of the Prognostic Signature

Having developed the immune-related lncRNA-based signature, the ROC curve was plotted and the AUC was calculated to check the efficacy of this signature. Besides, the multivariate time-dependent ROC curve was used to compare this signature with other commonly used clinicopathological characteristics like age, gender, stage, and grade. Also, the cutoff with the most AUC was regarded as the threshold to distinguish each sample as high/low risk. Then, the Kaplan-Meier was performed to plot the survival curve, and the log-rank test was used to test the survival differences between the risk strata. Following this, we wondered about the relation between risk strata and their clinicopathological characteristics and performed a chi-square test to check its clinical correlation. Besides, according to the clinical subgroups, we separately conducted survival analysis and performed Wilcoxon's signed-rank test to check the differential distribution of the risk score between clinical subgroups. Finally, we performed univariate and multivariate Cox's regression to check whether this prognostic signature could serve as an independent prognostic factor.

### 2.5. Immune Infiltration, Immune Checkpoint Expression, and Drug Response

There were several acknowledged methods to estimate the immune infiltration of samples according to their transcriptional atlas. Here, we conducted seven different methods to investigate the immune infiltration status of KIRC patients precisely, including XCELL, TIMER, QUANTISEQ, MCPCOUNTER, EPIC, CIBERSORT-ABS, and CIBERSORT. Then, we applied the SPEARMAN correlation test to explore the risk score that was significantly related to the infiltrating immune cells with *p* < 0.05.

Additionally, we wondered whether the immune checkpoints were differentially expressed between high-/low-risk patients; thus, we separately extracted the expression of PDCD1 (PD1), CD247 (PDL1), CTLA4, TIGIT, and LAG3, and then we compared their differential expression using the Wilcoxon signed-rank test.

Finally, the drug response of first-line targeted therapy for KIRC was evaluated by applying the R package “pRRophetic” to predict each patient's drug sensitivity to sunitinib. The drug sensitivity between high-risk and low-risk patients was compared using the Wilcoxon signed-rank test.

## 3. Results

### 3.1. Identification of Differentially Expressed irlncRNAs (DEirlncRNAs)

**Figure 1:**
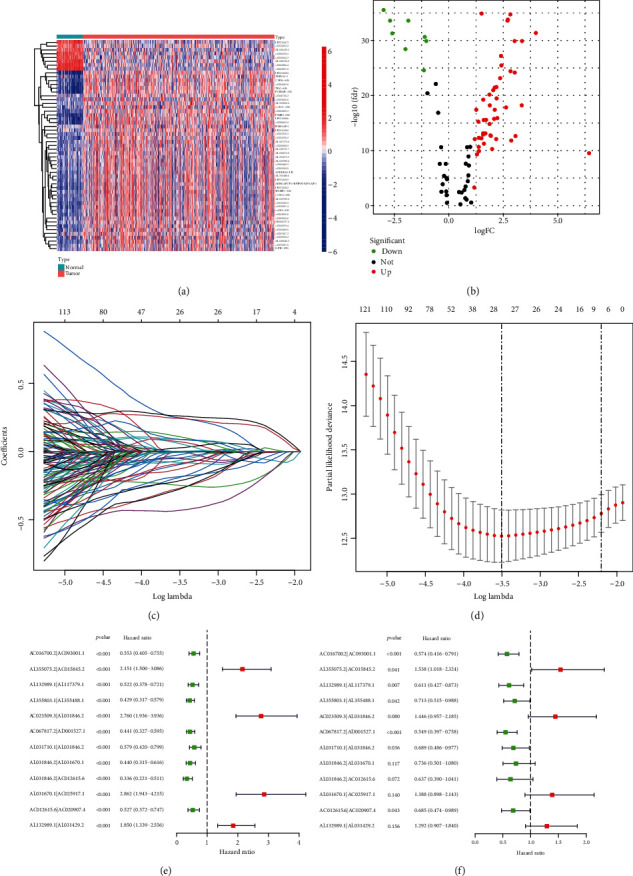
Establishment of a risk assessment model using DEirlncRNA pairs. Identification of differentially expressed immune-related lncRNAs (DEirlncRNAs) using TCGA datasets and annotation by Ensembl. (a and b) The heat map (a) and volcano plot (b) are shown. (c) Variables going to zero as we increase the penalty (lambda) in the objective function of the LASSO. (d) 10-fold cross-validation for tuning parameter selection in the LASSO model, −4 < lambda.min < −3.5, and there were 28 variables (immune-related lncRNA pairs) left. (e) The univariate Cox regression analysis of the 12 DEirlncRNA pairs. (f) A forest map shows 12 DEirlncRNA pairs identified by the Cox proportional hazard regression in the stepwise method.

First, we retrieved the kidney clear cell renal cell carcinoma (KIRC) project of The Cancer Genome Atlas (TCGA) database for the transcriptome profiling data of the kidney clear cell renal cell carcinoma and found 539 tumor samples and 72 normal samples. Then, we annotated the data according to Ensembl's gene transfer format (GTF) file and performed coexpression analysis between 2483 known ir-genes from IMMPORT and 13,162 lncRNAs after annotation. Finally, we found 95 irlncRNAs in total, among which 55 were classified as DEirlncRNAs (Figures 1(a) and 1(b)). Among these irlncRNAs, 47 were upregulated and 8 were downregulated (Figure 1(b)).

### 3.2. Establishment of DEirlncRNA Pairs and a Risk Assessment Model

**Figure 2:**
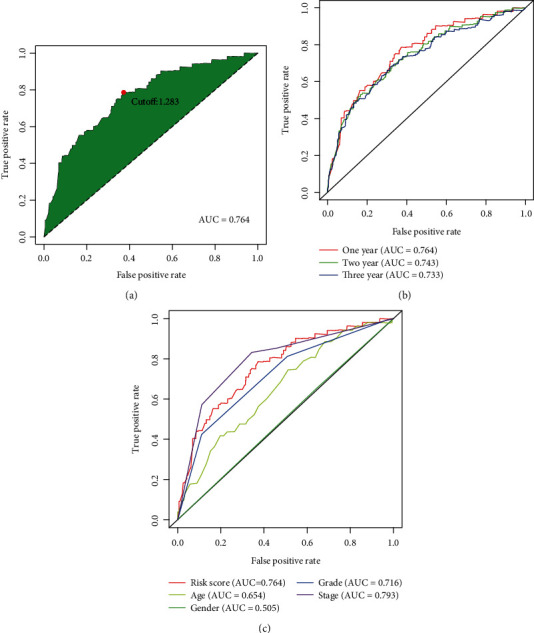
Establishment of a risk assessment model by DEirlncRNA pairs. (a) The ROC of the optimal DEirlncRNA pair models was related to the maximum AUC, and the cutoff point was calculated by the AIC. (b) The 1-, 2-, and 3-year ROC of the optimal model suggested that all AUC values were over 0.73. (c) A comparison of 1-year ROC curves with other common clinical characteristics.

We used an iterative loop and a 0-or-1 matrix and successfully sifted 918 valid DEirlncRNA pairs from 55 DEirlncRNAs. We adopted a single-factor test and a modified LASSO regression analysis to screen out 28 pairs of DEirlncRNAs (Figures 1(c) and 1(d)) and then constructed a multivariate Cox proportional hazard model using 12 pairs of them by a stepwise method (Figure 1(f), Table 1). The univariate Cox regression of these 12 lncRNA pairs are shown as Figure 1(e). Next, we drew the receiver operating characteristic (ROC) curve of all the 12 DEirlncRNA pairs and calculated the area under the curve (AUC), the maximum of which is 0.764 (Figure 2(a)). And this DEirlncRNA pair with the maximum AUC value was thought to be the optimal choice. Then, we drew the 1-year, 2-year, and 3-year ROC curves and found that all the values were greater than 0.73 (Figure 2(b)). Next, we plotted the ROC curves of the other clinical features like age, gender, grade, and stage and compared them with the ROC curve drawn by the DEirlncRNA pair, which showed that only the AUC of the ROC curve drawn by stage is greater than the one using the DEirlncRNA pair (Figure 2(c)). All these above have revealed that this model is reasonable and has a comparable clinical significance as the other clinical characteristics. Then, a cutoff point was found by using the Akaike Information Criterion (AIC) value in the one-year ROC curve (Figure 2(a)). Afterwards, we used the data of 530 appropriate ccRCC patients collected from TCGA and figured out the risk scores for all patients. Then, we used the cutoff value calculated above to divide the patients into two groups: the high-risk and low-risk groups, for further verification. The high-risk group included 220 cases, and the low-risk one included 310 cases (Table 2).

### 3.3. Clinical Evaluation by Risk Assessment Model

**Figure 3:**
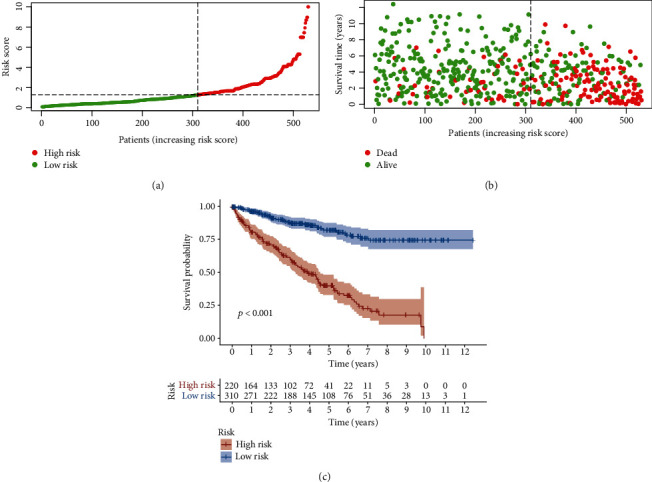
Risk assessment model for prognosis prediction. (a and b) Risk scores (a) and survival outcome (b) of each case are shown. (c) Patients in the low-risk group experienced a longer survival time tested by the Kaplan-Meier test.

**Figure 4:**
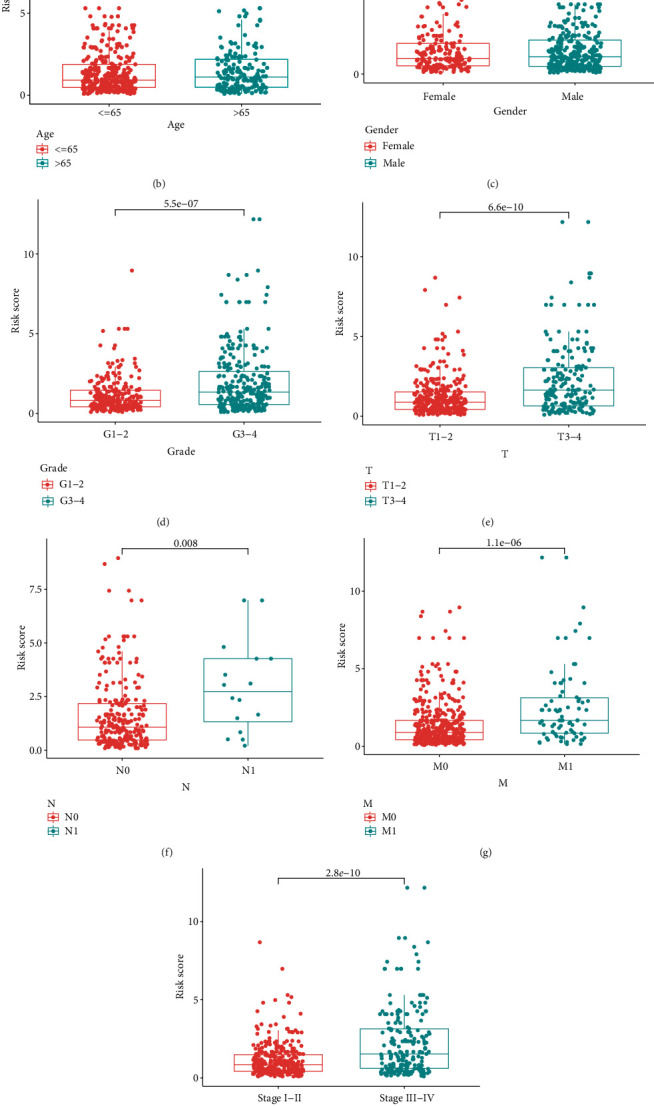
Clinical evaluation by the risk assessment model. (a–j) A strip chart (a) along with the scatter diagram showed that (b) age and (c) gender were not significantly associated with the riskScore but (d) tumor grade, (e) T stage, (f) N stage, (g) M stage, and (h) clinical stage were significantly associated with the riskScore. (i) A univariate Cox hazard ratio analysis demonstrated that age (*p* < 0.001; HR = 1.032; 95% CI (1.018–1.045)), tumor grade (*p* < 0.001; HR = 2.279; 95% CI (1.859–2.795)), clinical stage (*p* < 0.001; HR = 1.863; 95% CI (1.633–2.126)), and riskScore (*p* < 0.001; HR = 1.389; 95% CI (1.316–1.465)) were statistically different. (j) age (*p* < 0.001; HR = 1.035; 95% CI (1.021–1.050)), tumor grade (*p* = 0.004; HR = 1.394; 95% CI (1.111–1.750)), clinical stage (*p* < 0.001; HR = 1.534; 95% CI (1.316–1.789)), and riskScore (*p* < 0.001; HR = 1.301; 95% CI (1.224–1.384)) also showed statistical differences by the multivariate Cox regression analysis.

**Figure 5:**
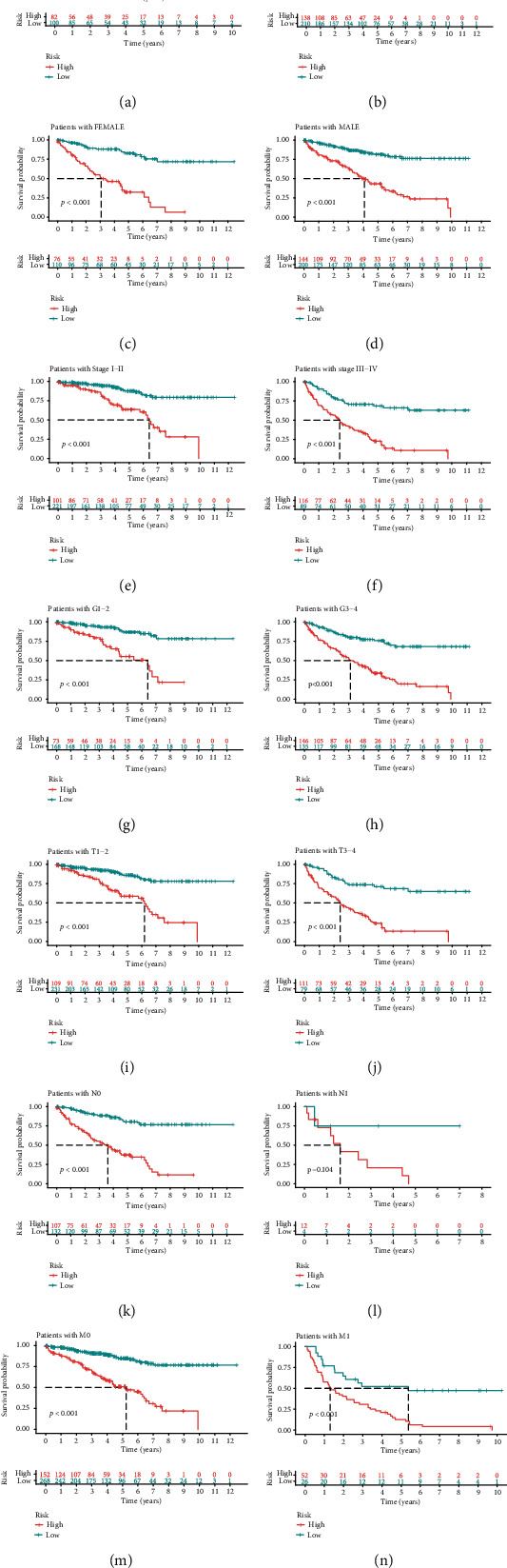
Subgroup survival analysis. (a) Risk score-based survival analysis in patients with age > 65. (b) Risk score-based survival analysis in patients with age ≤ 65. (c) Risk score-based survival analysis in female patients. (d) Risk score-based survival analysis in male patients. (e) Risk score-based survival analysis in patients with stages I and II. (f) Risk score-based survival analysis in patients with stages III and IV. (g) Risk score-based survival analysis in patients with G1-2. (h) Risk score-based survival analysis in patients with G3-4. (i) Risk score-based survival analysis in patients with T1-2. (j) Risk score-based survival analysis in patients with T3-4. (k) Risk score-based survival analysis in patients with N0. (l) Risk score-based survival analysis in patients with N1. (m) Risk score-based survival analysis in patients with M0. (n) Risk score-based survival analysis in patients with M1.

We calculated the risk scores for each patient, and they are shown in Figure 3(a). The survival time of each case is also displayed in Figure 3(b). These figures revealed that patients with low-risk scores would have a better clinical outcome than the ones with high-risk scores. Then, we plotted the survivorship curves for each group and used Kaplan-Meier's analysis to figure out if the difference had statistical significance. The outcome showed that the patients in the high-risk group had a shorter survival time than those in the low-risk group (*p* < 0.001) (Figure 3(c)). Next, we further explored the interaction between the risk of kidney clear cell renal cell carcinoma and several clinicopathological characteristics by using chi-square tests and acquiring a strip chart (Figure 4(a)). Then, we also performed a series of Wilcoxon's signed-rank tests and obtained several scatter diagrams, finding that tumor grade (Figure 4(d)), T stage (Figure 4(e)), N stage (Figure 4(f)), M stage (Figure 4(g)), and clinical stage (Figure 4(h)) interacted significantly with the risk, but age (Figure 4(b)) and gender (Figure 4(c)) were not significantly related to the risk. At last, we found that age (*p* < 0.001; HR = 1.032; 95% CI (1.018–1.045)), tumor grade (*p* < 0.001; HR = 2.279; 95% CI (1.859–2.795)), clinical stage (*p* < 0.001; HR = 1.863; 95% CI (1.633–2.126)), and riskScore (*p* < 0.001; HR = 1.389; 95% CI (1.316–1.465)) displayed statistical differences by the univariate Cox regression analysis (Figure 4(i)), and age (*p* < 0.001; HR = 1.035; 95% CI (1.021–1.050)), tumor grade (*p* = 0.004; HR = 1.394; 95% CI (1.111–1.750)), clinical stage (*p* < 0.001; HR = 1.534; 95% CI (1.316–1.789)), and riskScore (*p* < 0.001; HR = 1.301; 95% CI (1.224–1.384)) also showed statistical differences by the multivariate Cox regression analysis (Figure 4(j)). All these demonstrated that riskScore is a valuable prognostic predictor as other valid clinical predictors like tumor grade and clinical stage. Besides, it is interesting that among all the clinical subgroups, this signature performed well in distinguishing the great or poor outcome (Figures 5(a)–5(n)), including age > 65 or ≤65 (Figures 5(a) and 5(b)), female or male (Figures 5(c) and 5(d)), stages I and II or stages III and IV (Figures 5(e) and 5(f)), G1-2 or G3-4 (Figures 5(g) and 5(h)), T1-2 or T3-4 (Figures 5(i) and 5(j)), N0 or N1 (Figures 5(k) and 5(l)), and M0 or M1 (Figures 5(m) and 5(n)). These results showed the universality of our immune-related lncRNA pair-based prognostic signature.

### 3.4. Estimation of Tumor-Infiltrating Immune Cells and Immunosuppressive Molecules with Risk Assessment Model

**Figure 6:**
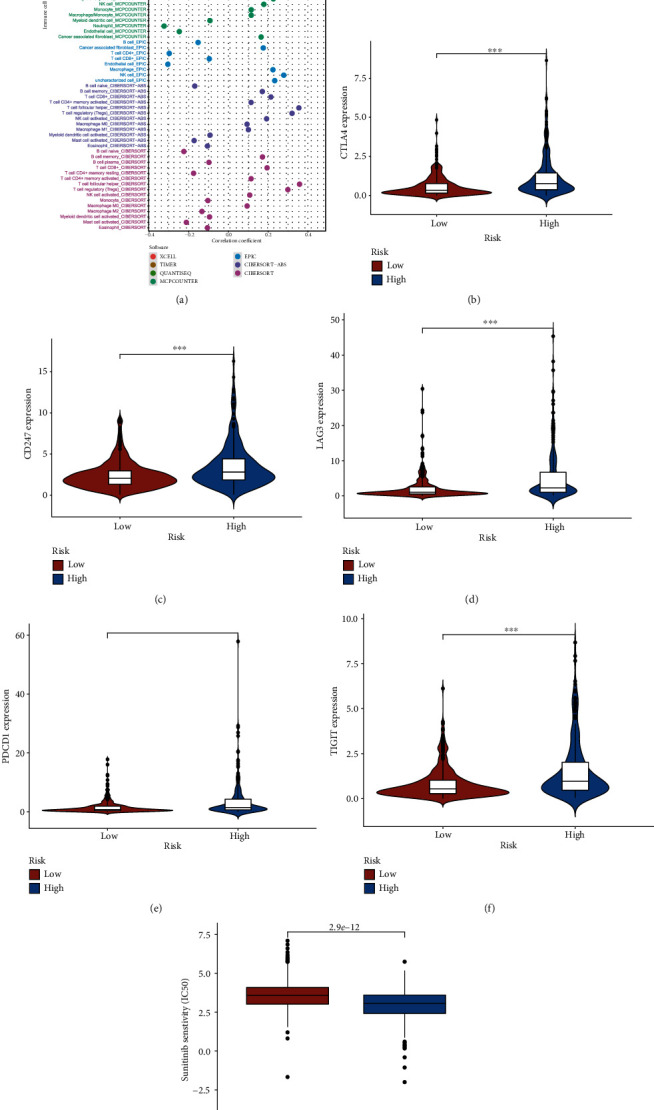
Estimation of tumor-infiltrating immune cells and immunosuppressive molecules with risk assessment model. (a) Patients in the high-risk group were more positively associated with Tregs as shown by the Spearman correlation analysis. (b–e) High-risk scores were positively correlated with upregulated (b) CTLA4 expression, (c) CD247 expression, (d) LAG3 expression, (e) PDCD1 expression, and (f) TIGIT expression levels, and all these differences had statistical significance in patients with ccRCC. (g) The model acted as a potential predictor for chemosensitivity as high-risk scores were related to a lower IC_50_ for chemotherapeutics such as sunitinib.

Since it was reported that lncRNAs may affect the tumor immune cell microenvironment by regulating tumor immune cell infiltration [14], we explored the relationship between the risk model and the tumor immune cell microenvironment in the next step. We performed a Spearman correlation analysis to obtain a lollipop shape (Figure 6(a)) and found that the riskScore was positively related with regulatory T cells (Tregs). Since immune checkpoint inhibitors are very popular in the treatment of kidney clear cell renal cell carcinoma, we screened out several targeted biomarkers critical for immune therapy and wanted to figure out whether or not the risk model was connected with them. We found that CTLA4 expression (Figure 6(b)), CD247 expression (Figure 6(c)), LAG3 expression (Figure 6(d)), PDCD1 expression (Figure 6(e)), and TIGIT expression (Figure 6(f)) were positively related to riskScore, and all these differences had statistical significance.

### 3.5. Analysis of the Correlation between the Risk Model and Chemotherapeutics

Targeted chemotherapeutics were more commonly used in the treatment of kidney clear cell renal cell carcinoma, so we wanted to dig out the interaction between the risk model and targeted agent sunitinib. We discovered that patients in the high-risk group had a lower half inhibitory centration (IC_50_) of sunitinib (*p* < 0.001) (Figure 6(g)), which means that the risk model had a latent predictive significance for the sensitivity of targeted chemotherapeutics.

## 4. Discussion

In recent years, more and more studies focus on the relationship between tumor and lncRNAs, and many lncRNA signatures have been established to predict the prognosis of tumor patients. Many previous lncRNA signatures are constructed based on several coding genes regulating the expression and modification of lncRNAs, or several lncRNAs regulating a certain biological process such as angiogenesis, autophagy, and ferroptosis, which have been proven to be connected with tumorigenesis and prognosis. A signature of nine coding genes regulating the methylation of the lncRNA promoter region has been established in patients with glioma [22]. Lei et al. [23], Tang et al. [24], and Li et al. [25] constructed lncRNA signatures related to angiogenesis, ferroptosis, and autophagy to predict the prognosis of hepatocellular carcinoma, head and neck squamous cell carcinoma, and breast cancer, respectively. Immune-related lncRNAs were also widely studied and included in many predictive models. For example, Chen et al. developed an immune-related seven-lncRNA signature for head and neck squamous cell carcinoma [26]. However, all these signatures are established based on the exact expression levels of lncRNAs. In this study, we used a model based on DEirlncRNA pairs composed of two related DEirlncRNAs, which has been formulated by Hong et al. [20] and is independent of their expression levels, and we adopted this novel model in kidney clear cell renal cell carcinoma for the first time and made some improvements. For example, we added a multivariate Cox proportional hazard model when sifting DEirlncRNA pairs and performing subgroup analyses for validation of the prognostic signature.

Primarily, we retrieved the kidney clear cell renal cell carcinoma (KIRC) project of The Cancer Genome Atlas (TCGA) database for the transcriptome profiling data of the kidney clear cell renal cell carcinoma and performed coexpression analysis between known ir-genes and lncRNAs to find DEirlncRNAs. Then, we used an iterative loop and a 0-or-1 matrix and sifted valid lncRNA pairs. Next, we adopted a single-factor test and a modified LASSO regression analysis to screen out ideal DEirlncRNAs and constructed a Cox proportional hazard model by a stepwise method. Afterwards, we calculated the AUC value of every ROC curve and found the optimal model, and then we compared this model with other ROC curves plotted by using other clinical characteristics such as gender, age, and stage to test the optimality. Then, a cutoff point was found by using the AIC value, and we distinguished the patients with kidney clear cell renal cell carcinoma into the high-risk group and the low-risk group using this value. Finally, we tested this model by investigating the relationship between risk score and survival, clinicopathological characteristics, cells in tumor immune microenvironment, chemotherapy, and targeted checkpoint biomarkers.

Many lncRNAs have been reported to participate in the tumorigenesis of renal carcinoma or have an influence on the prognosis of patients with malignant renal tumor. lncRNA HIF1A-AS2 was found to be connected with the malignant development and progression of renal carcinoma through the HIF1A-AS2-miR-30a-5p-SOX4 axis [27]. And lncRNA ZNF582-AS1 was reported to work as a tumor suppressor, which was downregulated in ccRCC and tightly related to the malignance of tumor, distant metastasis, and poor prognosis [28]. These studies all revealed that the exact expression levels of lncRNAs would have a latent predictive significance in clinical practice. However, measuring the exact expression levels of every lncRNAs is not always viable. In this article, we adopted a more concise model in which we used the combination of two lncRNAs instead of their specific expression levels. Thus, this model has stronger clinical practicability and can be applied on a more extensive scale. Moreover, some of the DEirlncRNAs we found in this article have never been reported before, such as AC016700.2 and AC093001.1, which may have a latent diagnostic and predictive significance and need further studies.

A tumor microenvironment is an ecosystem consisting of many kinds of adaptive and innate immune cells, which modulates the development and metastasis of all sorts of tumors [29]. Tumor-associated macrophages (TAMs) and T cells are the main constituents of the microenvironment [30]. T cells are the most abundant and feature-rich population in solid tumor TME. CD4+ T cells and CD8+ cells can prevent the tumor from developing by capturing tumor antigens and activating adaptive immunity to kill tumors [31]. However, the tumor cells will express immune checkpoint biomarkers such as PDL1 and CTLA4 to suppress T cell responses and lead to T cell exhaustion [32]. TAMs can be divided into two phenotypes, antitumor M1 and protumor M2 subtypes [33]. Tregs also participate in immune suppression and immune escape in TME. More and more studies have found that a large proportion of Treg cell infiltration in TME is connected with poor prognosis, and the removal of Treg cells in TME will enhance immune responses. Tregs highly express CTLA4 and can secrete immunosuppressive cytokines, which can suppress immune responses in TME and inactivate other immune cells [34]. ccRCC is one of the most immune-infiltrated tumors, in which the proportion of CD8+ T cells, Th1 T cells, dendritic cells (DC), and neutrophils is high, while the proportion of Th2 T cells and Tregs are relatively lower [35]. It is reported that PD1 is widely expressed in ccRCC, whereas the other inhibitory receptor such as TIM-3, CTLA4, and 4-1BB are only expressed in few PD1+ clusters, and CD38 is also widely expressed and may work as a latent T cell exhaustion marker [30]. So immune checkpoint inhibitors which block PD1/PDL1 or CTLA4 have been proven effective and considered as standard treatment [11, 36]. In our study, we have found that the risk score is positively related to Tregs, which is consistent with previous studies [37]. And we also found that CTLA4 expression, CD247 expression, LAG3 expression, PDCD1 expression, and TIGIT expression were positively related to the risk score, which means that this model has a potential predictive significance for the efficacy of immune checkpoint inhibitors.

However, we also realize that there still exist several shortcomings and limitations in our study. First, a large proportion of unknown lncRNAs were missing due to the intrinsic limitation of the microarray technique and probe repurposing method, and the original data for initial analysis were relatively limited and not general enough since they were simply downloaded from TCGA. And we are unable to obtain a data set that includes lncRNA expression levels, clinicopathological characteristics, and survival outcomes of ccRCC patients at the same time. Second, due to the difference in the expression levels of each sample which may make the final model unreliable, the constructed model requires external verification, although we have constructed a 0 or 1 matrix to screen all lncRNA pairs to minimize sample errors sourcing from expression changes and use various methods to test this model's optimality. Third, our study lacks verification from other clinical data sets which will be helpful to further confirm our model, and whether this model has a predictive significance in other types of cancer has not been verified. Therefore, we are prepared to collect some clinical samples to further verify our model, and assess the prognostic value of the lncRNA pair signature in other subtypes of RCC and other urinary system tumors. Fourth, we did not define the specific mechanisms in the connection between immune-related lncRNA pair signature and the prognosis of patients with ccRCC and the efficacy of immunotherapy, which should be further explored by laboratory experiments in the future.

In conclusion, in this study, we constructed a novel immune-related lncRNA pair signature in patients with ccRCC, which is based on the combination of two lncRNA pairs and independent of the exact expression level. And this model was proven to have a potential predictive significance for the prognosis of patients with ccRCC and the efficacy of immunotherapy.

## Figures and Tables

**Table 1:** Detailed information of the 12 immune-related lncRNA pairs included in the prognostic signature.

Id	Coef	HR	HR.95 L	HR.95H	*p* value
AC016700.2|AC093001.1	-0.555606139437541	0.573724398796137	0.416297695146405	0.790683421050959	0.000686076662003585
AL355075.2|AC015845.2	0.430419065848712	1.53790187067958	1.01755489950781	2.32433863272024	0.0410972283642356
AL132989.1|AL117379.1	-0.492662779184755	0.610997275328431	0.42746865065855	0.87332175092522	0.00686828997559175
AL355803.1|AL355488.1	-0.337782984347439	0.713350079234553	0.515147153257942	0.987811603588818	0.0419716042136084
AC023509.3|AL031846.2	0.368641620152125	1.44576937621004	0.956818971469132	2.18458156821172	0.0800537100889237
AC067817.2|AD001527.1	-0.600072662204743	0.548771759679331	0.397286721159376	0.758017895344506	0.000271616905474768
AL031710.1|AL031846.2	-0.372132850780792	0.689262667357912	0.486341990519643	0.976849693989471	0.0364731879647768
AL031846.2|AL031670.1	-0.306752711799432	0.735832541115505	0.501459754200406	1.07974672748735	0.1169244327575
AL031846.2|AC012615.6	-0.450512101167986	0.637301705094545	0.390057679070148	1.04126513874727	0.0720924820494324
AL031670.1|AC025917.1	0.327629977864943	1.38767540666321	0.898479178435956	2.14322499672168	0.139602853715524
AC012615.6|AC020907.4	-0.37879660475502	0.684684860149524	0.474231274929152	0.988533195723983	0.0432267830019594
AL132989.1|AL031429.2	0.255953118485003	1.29169217003435	0.906761769946673	1.84002978227255	0.156248628062543

**Table 2:** Clinicopathological characteristics of KIRC patients included in this study.

*n*	Overall	High risk	Low risk	*p*
	530	220	310	
Age (mean (SD))	60.56 (12.14)	61.08 (12.24)	60.20 (12.07)	0.409
Gender = female/male (%)	186/344 (35.1/64.9)	76/144 (34.5/65.5)	110/200 (35.5/64.5)	0.896
Grade (%)				<0.001
G1	14 (2.6)	3 (1.4)	11 (3.5)	
G2	227 (42.8)	70 (31.8)	157 (50.6)	
G3	206 (38.9)	93 (42.3)	113 (36.5)	
G4	75 (14.2)	53 (24.1)	22 (7.1)	
GX	5 (0.9)	1 (0.5)	4 (1.3)	
Unknown	3 (0.6)	0 (0.0)	3 (1.0)	
Stage (%)				<0.001
Stage I	265 (50.0)	76 (34.5)	189 (61.0)	
Stage II	57 (10.8)	25 (11.4)	32 (10.3)	
Stage III	123 (23.2)	61 (27.7)	62 (20.0)	
Stage IV	82 (15.5)	55 (25.0)	27 (8.7)	
Unknown	3 (0.6)	3 (1.4)	0 (0.0)	
T (%)				<0.001
T1	21 (4.0)	8 (3.6)	13 (4.2)	
T1a	140 (26.4)	35 (15.9)	105 (33.9)	
T1b	110 (20.8)	35 (15.9)	75 (24.2)	
T2	55 (10.4)	22 (10.0)	33 (10.6)	
T2a	10 (1.9)	5 (2.3)	5 (1.6)	
T2b	4 (0.8)	4 (1.8)	0 (0.0)	
T3	5 (0.9)	2 (0.9)	3 (1.0)	
T3a	120 (22.6)	71 (32.3)	49 (15.8)	
T3b	52 (9.8)	27 (12.3)	25 (8.1)	
T3c	2 (0.4)	2 (0.9)	0 (0.0)	
T4	11 (2.1)	9 (4.1)	2 (0.6)	
M (%)				<0.001
M0	420 (79.2)	152 (69.1)	268 (86.5)	
M1	78 (14.7)	52 (23.6)	26 (8.4)	
MX	30 (5.7)	16 (7.3)	14 (4.5)	
Unknown	2 (0.4)	0 (0.0)	2 (0.6)	
N (%)				0.004
N0	239 (45.1)	107 (48.6)	132 (42.6)	
N1	16 (3.0)	12 (5.5)	4 (1.3)	
NX	275 (51.9)	101 (45.9)	174 (56.1)	
riskScore (median (IQR))	1.03 (0.48, 2.01)	2.33 (1.66, 3.56)	0.54 (0.37, 0.87)	<0.001

## Data Availability

The datasets used and/or analyzed during the current study are available from the corresponding authors on reasonable request.
